# Integrating new and “old” cellular therapies in the evolving landscape of relapsed or refractory large B-cell lymphoma

**DOI:** 10.3389/fonc.2026.1924365

**Published:** 2026-09-14

**Authors:** Enrico Amaducci, Michele Clerico, Davide Camoirano, Claudia Marinucci, Luisa Giaccone, Irene Dogliotti, Michele Dicataldo, Mattia D’Agostino, Benedetto Bruno, Federica Cavallo

**Affiliations:** 1 Division of Hematology, Transplant and Cell Therapy Unit, Department of Molecular Biotechnology and Health Sciences, University of Torino, Turin, Italy; 2 Division of Hematology, Transplant and Cell Therapy Unit, Azienda Ospedaliero Universitaria (A.O.U) Città della Salute e della Scienza di Torino, Turin, Italy; 3 Department of Molecular Biotechnology and Health Sciences, University of Torino, Turin, Italy

**Keywords:** CAR T-cell therapy, hematopoietic stem cell transplant (HSCT), large B cell lymphoma, new therapeutic agents, secondary central nervous system lymphoma, transplantation, B-cell

## Abstract

The therapeutic algorithm for relapsed or refractory large B-cell lymphoma (R/R LBCL) is rapidly evolving. Pivotal Phase III trials, ZUMA-7 and TRANSFORM, have established second-line CD19-directed CAR T-cell therapies (axi-cel and liso-cel) as standard of care for primary refractory or early-relapsing disease, displaying superior survival outcomes compared with salvage chemoimmunotherapy followed by autologous hematopoietic stem cells transplantation (auto-HSCT) which is now limited to late relapses. However, clinical management of R/R LBCL becomes highly complex in peculiar settings, especially in case of multiple relapses even after CAR T-cell therapy, as described in our clinical case of secondary central nervous system disease relapse. Allogeneic HSCT remains a niche consolidative strategy exclusively for fit patients lacking other therapeutic options. Moving forward, novel bioengineering approaches, including dual-targeting, memory-enriched and “armored” CARs, aim to overcome current resistance mechanisms and redefine future clinical practice.

## 1 Introduction

The therapeutic landscape of relapsed/refractory (R/R) Large B-cell lymphoma (LBCL) has been fundamentally revolutionized by chimeric antigen receptor (CAR) T-cell therapies. Pivotal trials have successfully challenged the historical transplantation paradigm, establishing cellular therapy with CAR T-cells as the new standard of second-line treatment for high-risk R/R disease. Prompted by a complex clinical case of isolated central nervous system (CNS) relapse, this review begins evaluating approved second-line CAR products then outlining the restricted indications for both autologous and allogeneic stem cell transplantation (allo-HSCT) in the modern era and discuss how next-generation CAR engineering constructs aim to overcome resistance and redefine future clinical practice.

## 2 Clinical case: an early SCNSL post-CAR relapse treated with salvage allogeneic stem cell transplantation

A 43-year-old woman was diagnosed in October 2023 with diffuse large B-cell lymphoma (DLBCL), stage IVB, IPI 2 presenting with bulky right lateral cervical lymphadenopathy, likely representing transformation from a previous marginal zone lymphoma treated with single-agent rituximab in 2019. The patient received six cycles of R-CHOP, achieving a complete metabolic response at the end-of-treatment evaluation. Three months later, she experienced an early relapse with confluent FDG uptake in the right cervical lymph nodes, spleen, bones, and abdomen, suggestive of systemic disease involvement. Bridging therapy with two cycles of rituximab-polatuzumab was administered, followed by second-line CAR T-cell therapy with axicabtagene ciloleucel in September 2024. A new complete metabolic response was documented one month post-infusion. However, at 3.5 months she showed new-onset of headache, limb tremors with postural instability, and rapidly progressive weakness, culminating in partial paraplegia of the lower extremities. Disease progression with both systemic (pharyngeal disease) and central nervous system (D12–S1 spinal cord infiltration with leptomeningeal disease) was documented by lumbar puncture with detection of a CD19-positive B-cell population with light chain restriction, PET-CT scan and MRI confirming secondary central nervous system lymphoma (SCNSL) relapse. She proceeded with salvage therapy, including debulking radiotherapy with a single 8 Gy fraction, followed by MATRix regimen. Additionally, two intrathecal chemotherapy injections with cytarabine, dexamethasone and methotrexate were performed, resulting in improvement of neurological symptoms. Despite significant potential toxicities, the therapy was reasonably well tolerated and the hospitalization was complicated only by grade 2 intestinal mucositis. Restaging MRI after the third cycle of MATRix demonstrated a partial response, while the PET scan suggested a complete metabolic response. Given the high-risk disease course after CAR T failure, consolidation with allo-HSCT from an HLA-identical sibling donor was performed in May 2025, following reduced-intensity conditioning (RIC) with fludarabine, melphalan and total marrow and lymphoid irradiation (TMLI). At one year post-transplant, the patient remains in complete remission with stable clinical and hematologic parameters.

In this scenario, relapse localization after CAR limited the feasibility of subsequent treatment with bispecific antibodies (BsAbs), which are not yet reimbursed in our country for this indication. Based on the results of the MARIETTA trial, sequential MATRix combined with rituximab, ifosfamide, carboplatin, etoposide (MATRix-RICE) followed by autologous HSCT is currently considered the treatment of choice for CNS-directed therapy in patients with secondary CNS lymphoma achieving 1-year progression-free survival (PFS) of 58% (95% CI: 55–61), with even more favorable outcomes (2-year PFS up to 71%) observed in patients with CNS involvement at initial lymphoma diagnosis (1). Given the predominant localization to the CNS at relapse, we opted to limit salvage treatment to CNS-directed therapy alone, without the addition of systemic-intensity regimens. As soon as response was achieved, we proceeded directly to consolidation with allogeneic HSCT, considering the patient’s age, fitness and the response gained as favorable factors supporting this strategy.

## 3 CAR T-cell therapy in LBCL

### 3.1 Current utilization in second line and selection of CAR constructs

CAR T-cell therapy has revolutionized the management of high-risk relapsed or refractory LBCL in the second-line setting. Currently, two constructs are approved for patients relapsing within 12 months after frontline chemoimmunotherapy: axicabtagene ciloleucel (axi-cel) and lisocabtagene maraleucel (liso-cel). Main differences between ZUMA-7 and TRANSFORM trials regarded their clinical design: bridging therapy was prohibited in ZUMA-7 but permitted in TRANSFORM, where outpatient CAR T administration was also adopted. The studies also differed in terms of treatment crossover upon disease progression in the standard-of-care arm, which was allowed in TRANSFORM but not allowed in ZUMA-7 (2–5). The efficacy in second-line of CAR T-cell therapy has been also successfully demonstrated compared with the standard of care in patient ineligible for transplant (6–8). Although cross-trial comparisons should be interpreted cautiously, matching-adjusted indirect comparison (MAIC) analyses have reported comparable efficacy across products with respect to event-free survival, progression-free survival, and overall survival (9). Nevertheless, distinct expansion kinetics and toxicity profiles have been observed. From a safety perspective, liso-cel showed lower Cytokine Release Syndrome (CRS) rate: any-grade and severe (≥ grade 3) CRS dropped from 92%/6% with axi-cel to 49%/1% with liso-cel, while any-grade and severe Immune Effector Cell-Associated Neurotoxicity Syndrome (ICANS) decreased from 60%/21% to 12%/4%, respectively (2, 4, 10). This clinical variance is biologically driven by their co-stimulatory domains: axi-cel incorporates a CD28 co-stimulatory domain, resulting in rapid *in vivo* expansion with peak CAR T-cell levels typically occurring around 7 days after infusion versus 12 days per liso-cel (2, 4). This characteristic, along with a shorter manufacturing time for axi-cel (11), may be advantageous in patients with highly aggressive or rapidly progressive disease but is also associated with a higher incidence of CRS and ICANS (12). In contrast, liso-cel utilizes a 4-1BB co-stimulatory domain and is manufactured with a balanced CD4+/CD8+ cellular composition, promoting more sustained expansion and persistence while maintaining a favorable safety profile (4, 13).

Depending on disease kinetics, holding therapy may be administered before lymphocyte apheresis to control tumor burden, while bridging therapy may be given between apheresis and CAR T-cell infusion to prevent disease progression during the manufacturing period. Both strategies require adherence to appropriate wash-out periods; the use of bendamustine is not recommended prior to leukapheresis, particularly within a critical cut-off of 9 months before as recent exposure has been associated with worse response rates and survival (14). Although data evaluating the impact of bridging therapy on survival outcomes remain controversial (15–17), this strategy is often necessary and indicated in the context of aggressive disease to improve patient symptoms or to control rapidly progressive disease. It is also true that studies in the literature document a correlation between pre-infusion Eastern Cooperative Oncology Group (ECOG) performance status, LDH levels and tumor burden (expressed as metabolic tumor volume) and survival (18, 19). The strategies used mainly include chemo-immunotherapy approaches or radiotherapy. One of the most widely used regimens is polatuzumab vedotin, rituximab, and bendamustine (pola-BR) which offers good response rates with limited toxicity (15, 20, 21). In patients who are not eligible for chemotherapy or who have less aggressive disease the omission of bendamustine may be considered in order to limit toxicity while maintaining satisfactory response rates (21). For patients with localized disease, radiotherapy represents a viable option, with high response rates, minimal toxicity (22, 23), and evidence of immunomodulatory effects that positively impact the efficacy of subsequent CAR T-cell therapy (24, 25).

With regard to CAR T-cell therapy in SCNSL, the pivotal trials leading to approval of the three commercially available anti-CD19 constructs, axi-cel, tisagenlecleucel (tisa-cel), and liso-cel, largely excluded patients with active CNS involvement, owing to concerns regarding an increased risk of neurotoxicity. However, real-world evidence has since helped fill this gap: the French registry, the largest cohort to date (n=195), included patients treated predominantly with axi-cel (71%), followed by liso-cel (16%) and tisa-cel (13%), and reported 12- and 18-month PFS rates of 46% (95% CI: 37-54) and 37% (95% CI: 27-47), respectively, with no major differences in efficacy across the three products (26). These findings support anti-CD19 CAR T therapy as a feasible and effective option in SCNSL, irrespective of the specific construct employed.

### 3.2 Post CAR T-cell failure scenario and underlying resistance mechanisms

Although second-line CAR T-cell therapies have substantially improved outcomes in R/R LBCL, a significant proportion of patients ultimately experience relapse or disease progression. Resistance is driven by a complex interplay between tumor-related mechanisms, the tumor microenvironment (TME) and CAR T-cell dysfunction (27–29). Antigen escape is one of the most common tumor-intrinsic mechanisms of failure. Under selective immune pressure, lymphoma cells may downregulate or lose expression of the target antigen through alternative splicing, genetic alterations, lineage plasticity or trogocytosis, thereby reducing CAR T-cell recognition and cytotoxicity (30–37). In our case, flow cytometric analysis of the cerebrospinal fluid at relapse identified a CD19-positive lymphoma population, indicating that antigen loss was not the underlying mechanism of treatment failure. The TME contributes to treatment resistance by promoting an immunosuppressive state characterized by immune checkpoint activation (38, 39), accumulation of regulatory immune populations, and secretion of inhibitory cytokines (37, 40). Myeloid-derived suppressor cells (41–44), regulatory T cells, and tumor-associated macrophages (TAMs) (45–47) impair CAR T-cell expansion and function, while metabolic alterations further compromise antitumor activity (48, 49). A further challenge is represented by CAR T-cell exhaustion: sustained antigen exposure induces progressive functional impairment, characterized by reduced proliferative capacity, diminished cytokine production, and increased expression of inhibitory receptors, including PD-1, TIGIT, TIM-3, and LAG-3 (50–54). Collectively, these mechanisms limit CAR T-cell persistence and contribute to loss of durable disease control. T-cell/histiocyte-rich large B-cell lymphoma (TCRLBCL) population represents a prime example of how the expression of PD-L1, both on clonal cells and within the TME (e.g. PD-L1 expressing TAMs) can interfere with CAR T-cell killing resulting in a high incidence of disease progression (55, 56).

From a clinical standpoint, managing patients who progress after CAR T therapy represents an unmet medical need with historically poor outcomes (57–59). Current post-failure strategies are highly heterogeneous and dependent on the patient’s performance status, including salvage chemotherapy, targeted agents (loncastuximab tesirine) (60), bispecific antibodies (epcoritamab or glofitamab) (61), immune-checkpoint inhibitors (CPIs) (62, 63) or enrollment in clinical trials (64, 65) evaluating next-generation CAR constructs and other molecules. In eligible, highly selected patients who achieve a subsequent response, non-myeloablative allo-HSCT remains a potential consolidative strategy with curative intent.

## 4 “Old” cellular therapies and their application

### 4.1 Role of autologous stem cell transplantation in first relapse

Historically, high-dose therapy followed by autologous stem cell transplantation (auto-HSCT) was the gold standard for all R/R LBCL patients responsive to salvage chemotherapy (66). However, its efficacy is highly dependent on the timing of relapse, demonstrating dismal results in high-risk patients with primary refractory disease or early relapse occurring within 12 months (67). The trials evaluating second-line CAR therapies versus standard of care (SOC) provided definitive quantification of this therapeutic failure. In the ZUMA-7 trial, only 36% of patients randomized to the standard auto-HSCT arm actually managed to receive the transplant, primarily due to early disease progression during salvage chemotherapy (2); similarly, in the TRANSFORM trial, only 47% of patients in the SOC arm successfully proceeded to auto-HSCT consolidation (4). Furthermore, for patients treated with auto-HSCT, the median event-free survival (EFS) remained significantly inferior in the longer-term follow-up analysis of both trials. In the primary survival analysis of ZUMA-7 (median follow-up 47.2 months), investigator assessed median EFS was 10.8 months with axi-cel versus 2.3 months with SOC. Similarly, in the 3-year follow-up of TRANSFORM median EFS was 29.5 months with liso-cel versus 2.4 months with SOC. EFS was the primary endpoint in both trials, and the control-arm EFS values refer to the intention-to-treat (ITT) analysis of the entire SOC arm rather than to the specific subgroup of patients who actually underwent auto-HSCT (3, 5).

Conversely, data suggest that auto-HSCT could maintain a therapeutic rationale exclusively within a highly selected niche of late-relapsing patients whose disease recurs more than 12 months from effective frontline therapy (64, 65). In this favorable subgroup, auto-HSCT still yields long-term durable remissions, particularly in patients who achieve response after a single line of salvage therapy: in a retrospective cohort of 151 patients with DLBCL relapsing ≥12 months, those requiring only one line of salvage therapy had markedly superior outcomes compared with patients requiring more than one line (median PFS 61.4 vs. 6.1 months, median OS 111.7 vs. 17.8 months). Patients achieving CR after a single line of salvage therapy had an even more favorable median PFS of 63.7 months post-ASCT, whereas those requiring additional salvage lines should be considered for alternative treatment strategies (68).

Notably, the therapeutic armamentarium is expected to expand shortly with the upcoming availability of the glofitamab or epcoritamab plus GemOx (gemcitabine and oxaliplatin) combination (69, 70) in this setting further challenging the role of autologous transplantation in R/R LBCL.

### 4.2 Positioning of allogeneic stem cell transplantation as salvage treatment

The role of allo-HSCT in R/R LBCL is currently confined to highly selected clinical scenarios, shifting from its historical role as an early consolidation strategy to a delayed post-CAR salvage strategy (71). This change reflects the favorable safety profile of CAR T-cells, including the absence of graft-versus-host disease (GvHD), lower non-relapse mortality (NRM), and fewer long-term complications compared with allo-HSCT, although the durability of responses and the efficacy of salvage strategies other than allo-HSCT remain to be further outlined (72). Importantly, CAR T-cell therapy is also effective in patients with active, refractory disease, whereas allo-HSCT usually requires prior objective, ideally complete response, a challenging prerequisite in the context of multiple treatment failures. Thus, in the current era, allo-HSCT is generally considered after failure of CAR T therapy (73), as illustrated in our case-report. Bispecific antibodies, ADCs and other novel agents or combinations can induce remissions in chemo- and CAR-refractory patients, potentially reaffirming allo-HSCT role as consolidation strategy now that CAR T-cell therapy has moved to second-line setting.

The primary therapeutic rationale for an allogeneic approach relies on the immunological graft-versus-lymphoma (GVL) effect to eradicate residual clones that have developed absolute resistance to both conventional chemotherapy and novel CAR T-cell constructs (74). In current clinical practice, this high-risk procedure, still characterized by significant NRM, is reconsidered almost exclusively for young, fit patients who maintain an optimal performance status (75). Disease status at the time of transplant is the central determinant of outcome: achievement of a complete remission (CR) prior to conditioning represents the strongest independent predictor of post-allogeneic transplant success, whereas active or rapidly progressive disease at stem cell infusion, or exposure to multiple lines of therapy without achieving CR, consistently compromises survival (72, 73, 75, 76). Allo-HSCT currently represents the only potentially curative option in the event of BsAb salvage failure; however, the role of allo-HSCT consolidation in patients achieving CR after BsAb treatment remains controversial (72).

In heavily pretreated patients, choice of conditioning regimen needs to be carefully balanced, possibly with a multidisciplinary approach to identify potential toxicities from previous chemoimmunotherapies or radiation therapy. For instance, accurate cardiac function evaluation needs to be performed immediately prior to allo-HSCT to rule out cardiotoxicity, particularly in patients previously exposed to anthracyclines; pulmonary function tests are also critical in patients who received previous radiotherapy or suffered from lung infections during previous chemotherapy lines (77). The use of RIC regimens is generally preferred to reduce further accumulation of toxicities (78), given the fact that the efficacy of allo-HSCT is mainly related to GVL effect rather than conditioning intensity in such patients. Novel approaches such as TMLI protocols might also be evaluated if available and according to local guidelines (79). Immunosuppression levels need to be accurately balanced, with the aim of reducing relapse and GvHD risks while enhancing GVL effect. In order to achieve these goals, patients need to be carefully monitored for acute and chronic GvHD occurrence. Tapering of immunosuppression should begin as soon as possible in the absence of GvHD signs or symptoms, generally starting from two/three months after transplantation, depending on disease state at transplant and individualized relapse risk (80). Post-transplant cyclophosphamide (PTCy) use might help reduce the risk of GvHD and can be considered according to local practice (81). Survival outcomes reported in the few studies of patients undergoing allo-HSCT after failure of CAR T-cell therapy have generally been poor, with median PFS ranging from 4 to 10 months and median OS reaching approximately 20 months (**Table 1**). Notably, the cohorts summarized in **Table 1** include only patients who survived, responded to salvage therapy and had a suitable donor available, a selection process that warrants consideration when interpreting the reported outcomes.

**Table 1:** Allo-HSCT after CAR-T in LBCL cohorts from key studies.

Authors	Barone et al.	Zurko et al.	Derigs et al.	Fried et al.
Year	2025	2023	2023	2023
N° post-CAR R/R pts	83	NA	214 (143 with FU data)	NA
N° alloHSCT pts	13	88	40	39
Histology	DLBCL (61%), tDLBCL (31%), PMBCL	DLBCL (59%), tNHL (26%), PMBCL (9.1%), HGBCL NOS (5.7%)	LBCL	DLBCL (77%), HGBCL (13%), PMBCL (10%)
Double/triple hit	NA	12%	NA	22%
Median n° of prior lines between CAR-T to allo-HSCT	5 (3-9)	1 (0-7)	NA	NA
Median time from CAR-T to failure of allo-HSCT	8.9 months (2.3-21.9)	92 days (7-527)	103 days (21-366)	127 days (48-661)
Salvage therapy after CAR-T failure	Glofitamab (100%)	CHT (18%), Pola-based (18%), RT (13%), CPI (11%), CAR-T/NK cell therapy (10%)	Pola-based, RT, CHT, ibrutinib-based, CPI-based, lenalidomide-based*	Pola-based (28%), CHT (13%), BTKi-based (13%), RT, lenalidomide-based, BsAb, CPI, allogeneic CAR-T
Disease status at transplantation	CR 11 (85%), PR 2 (15%)	CR 45 (51%), PR 22 (25%), SD/PD 21 (24%)	CR 8 (20%), PR 14 (35%), SD 3 (8%), PD 11 (28%), untreated relapse 4 (10%)	CR 16 (41%), PR 15 (38%), PD 8 (21%)
Donor	MRD 2 (16%), MUD 5 (38%), haplo 6 (46%)	MUD (39%), haplo (30%), MRD (25%), MMUD (3%), cord (2%)	NA	MSD (36%), MUD (36%), others (28%)
Stem cell source	PBSC (100%)	PBSC (86%), BM (11%), cord (2%)	NA	PBSC (100%)
Conditioning regimen	FluCyTBI2Gy 5 (38.5%), ThioFluCy 5 (38.5%), TBF 3 (23%)	MAC (23%), RIC (77%)	NA	MAC (3%), int-intensity (38%), low-intensity (59%)
GvHD prophylaxis	CsA+MMF 8 (62%), CsA+MTX 5 (38%)+ATG 2 or MMF 2	CNI+MTX (25%), TAC/MMF/PTCY (49%), other (26%)	NA	CNI+MTX (69%), CNI+MMF (31%)
Factors associated with survival	NA	≥2 lines between CAR-T and allo-HSCT, PR at allo-HSCT, hispanic	Responsive disease (p=.09)	LDH, transformed lymphoma, HCT-CI ≥3, PD at allo-HSCT
PFS	mPFS: 4 months (ITT)	mPFS: 10 months	NA	2y-PFS: 31%
OS	NR	mOS: 21 months (95% CI: 12 months-NR)	1y-OS: 36%	2y-OS: 45%
NRM	0%	1y-NRM: 22%	NA	2y-NRM: 26%
Incidence of relapse or progression	0% (median FU 18.4 months)	1y 33% (95% CI: 24-45)	NA	43% (95% CI: 27-59)
aGvHD	6 pts (46%), G1-2: 5, G3-4: 1	G2-4: 34%, G3-4: 10%	NA	G2-4: 38.5%, G3-4: 5.4%
cGvHD	4 (30%), mild 2, moderate 2	Mild 16%, moderate 7.8%, severe 3.8% at 1 year	NA	Moderate-severe at 2 year: 11.5%
Infections	Bacterial infections <100d 5 (39%)	NA	NA	36%

*in patients candidate and non-candidate to allo-HSCT.

N°, number; CAR, chimeric antigen receptor; R/R, relapsed or refractory; pts, patients; FU, follow-up; allo-HSCT, allogeneic hematopoietic stem cell transplantation; DLBCL, diffuse large B-cell lymphoma, tDLBCL, transformed diffuse large B-cell lymphoma; PMBCL, primary mediastinal large B-cell lymphoma; tNHL, transformed non-Hodgkin lymphoma; HGBCL NOS, high grade B-cell lymphoma not otherrwise specified; LBCL, large B-cell lymphoma; CHT, chemotherapy; pola, polatuzumab-vedotin; RT, radiotherapy; CPI, check-point inhibitor; BTKi, Bruton’s tyrosine kinase inhibitor; CR, complete respone; PR, partial response; SD, stable disease; PD, progressive disease; MRD, matched related donor; MUD, matched unrelated donor; haplo, haploidentical; MMUD, mismatched unrelated donor; MSD, matched sibling donor; PBSC, peripheral blood stem cell; BM, bone marrow; flu, fludarabine; Cy, cyclophosphamide; TBI, total body irradiation; Gy, gray; thio, thiotepa; TBF, thiotepa, busulfan, fludarabine; MAC, myeloablative conditioning; RIC, reduced intensity conditioning; int, intermediate; GvHD, graft versus host disease; CSA, cyclosporine; MMF, mycophenolate mofetil; MTX, methotrexate; ATG anti-thymocyte globulin; CNI, calcineurine inhibitor; TAC, tacrolimus; PTCY, post-transplant cyclophosphamide; NA, non available; LDH, lactate dehydrogenase; HCT-CI, Hematopoietic Cell-Transplantation-specific Comorbidity Index; PFS, progression free survival; mPFS, median progression free-survival; ITT, intention-to-treat; 2y, 2 years; OS, overall survival; NR, not reached; mOS, median overall survival; CI, confidence interval; 1y, 1 year; NRM, non-relapse mortality; FU, follow-up; aGvHD, acute graft versus host disease, cGvHD, G1-2, grade 1 and 2; G2-4, grande 2 and 4; G3-4, grade 3 and 4; chronic graft versus host disease; 100d, day 100.

Overall, while the rapid expansion of CAR T-cell therapy and other off-the-shelf immunotherapies has significantly narrowed its historical indications, allo-HSCT remains a potentially curative consolidative option for this specific subgroup of responsive patients, provided that the potential for long-term disease control substantially outweighs the non-negligible risks of transplant-related mortality and GvHD (**Table 1**).

## 5 Novel CAR T-cell constructs and emerging data

To overcome the current limitations of approved CAR T-cell products, mainly driven by antigen escape and limited long-term CAR persistence, next-generation cellular therapies are rapidly evolving through novel bioengineering strategies and are currently being evaluated in clinical trials for R/R LBCL. Current strategies can be broadly classified into complementary approaches: dual-target antigen recognition, preservation of T-cell stemness and accelerated manufacturing, modulation of the tumor microenvironment (82) (**Table 2**).

**Table 2:** Overview of novel CAR-T cell products.

CAR-T construct	Trial (phase)	N° pts	Current therapy line	Histology	Target(s)	Co-stimulatory domain	Manufacturing time	Fresh vs cryopreserved	T-cell stemness preservation	Lymphodepletion regimen	ORR (%)	CRR (%)	Any-grade CRS	Grade ≥3 CRS	Any-grade ICANS	Grade ≥3 ICANS	Other toxicities		Median follow-up (months)
Zamtocabtagene autoleucel	II	82 (zamto-cel), 86 (SoC)	2L	DLBCL (85%)	CD20-CD19	4-1BB	12 days 15 days (VTV)	Fresh	Rapid manufacturing	Flu-Cy	72	54	NA	5.3%	NA	1.3%	G4 persisting neutropenia (9%)	17	
Rondecabtagene autoleucel	I/II	60	2L or 3L+	LBCL	CD20-CD19	4-1BB	16 days (VTS)	Cryopreserved	From CD62L+ and Tcm cells	Flu-Cy	87 (2L) 90 (3L+)	60 (2L) 71 (3L+)	62% (G1-2)	0	12% (G1-2)	13%	/	7 and 10	
Prizloncabtagene autoleucel	I	53	2L+	DLBCL (77.1%)	CD20-CD19	4-1BB	22 days (VTV)	NA	No	Flu-Cy	91.5	85.1	93.8%	1 pt	6.3%	0	Neutropenia (83.3%)	30	
KITE-363	I	37	2L+ (LBCL) or 3L+	LBCL, iNHL, NLPHL, LBCL/classical HL	CD20-CD19	CD28 (anti-CD19 CAR), 4-1BB (anti-CD20 CAR)	NA	NA	No	Reduced	87 (CAR naïve)	78 (CAR naïve)	NA	1 pt	NA	3 pts	/	11	
KITE-753	I	14	2L+ (LBCL) or 3L+	LBCL (71%) iNHL (29%)	CD20-CD19	CD28 (anti-CD19 CAR), 4-1BB (anti-CD20 CAR)	NA	NA	Rapid manufacturing	NA	64	100 (DL3) 45 (DL1,2)	3 pts (G1-2)	1 pt	2 pts	0	/	4.4	
GLPG5101	I/II	24	2L+	DLBCL	CD19	4-1BB	7 days (VTV)	Fresh	Rapid manufacturing	Flu-Cy	75 (ITT) 82% (EAS) 90% (3L+)	58% (ITT) 64% (EAS) 80% (3L+)	41%	1 pt	27% (all G1)	1 pt	/	15.1	
Rapcabtagene autoleucel	II	37	1L	LBCL	CD19	4-1BB	<2 days	Cryopreserved	Rapid manufacturing	Flu-Cy	90	74	38% (G1-only)	0	8%	NA	1 IEC-HS	4.2	
huCART19-IL18	I	21 (20 CAR-exposed)	4L+	LBCL (12 pts) FL (6 pts) MCL (3 pts)	CD19	4-1BB	3 days	Cryopreserved	Rapid manufacturing	Flu-Cy Bendamustine	81	52	62%	14%	14%	0	/	17.5	

CAR chimeric antigen receptor; N° pts number of patients; ORR overall response rate; CRR complete response rate; CRS Cytokine Release Syndrome; ICANS Immune Effector Cell-Associated Neurotoxicity Syndrome; L line; DLBCL diffuse large B-cell lymphoma, LBCL large B-cell lymphoma; iNHL indolent non-Hodgkin lymphoma; HL Hodgkin lymphoma; FL follicular lymphoma; MCL mantle cell lymphoma; VTV vein-to-vein; VTS vein-to-site; NA non-available; Flu fludarabine; Cy cyclophospamide; ITT intention-to-treat; EAS efficacy analysis set; G grade; IEC-HS immune effector cell-associated hemophagocytic lymphohistiocytosis-like syndrome.

### 5.1 Dual target CAR T-cell constructs

Targeting multiple antigens simultaneously represents one of the most promising strategies to prevent immune escape following CD19-directed therapy. Most dual-target constructs combine CD19 and CD20 recognition through bicistronic, tandem or OR-gate designs, thereby reducing the likelihood of antigen-negative relapse while maintaining broad anti-lymphoma activity. Although currently supported predominantly by early-phase trials, dual-target constructs have consistently demonstrated encouraging response rates with manageable toxicity profiles. Several constructs are currently under clinical investigation. Zamtocabtagene autoleucel (zamto-cel), rondecabtagene autoleucel (ronde-cel), prizloncabtagene autoleucel (prizlon-cel), KITE-363 and KITE-753 all exploit dual CD19/CD20 targeting but differ substantially in manufacturing platforms and CAR architecture (83–88). These constructs demonstrate promising response rates, with ORR ranging from 60% to 90.7% and CR rates from 42% to 85% (**Table 2**), as shown predominantly in Phase I trials enrolling limited numbers of R/R patients. Toxicity data indicate a favorable safety profile across all constructs, with few episodes of grade ≥3 CRS or ICANS. Ronde-cel has progressed furthest in clinical development, the ongoing Phase III PINACLE-H2H trial (clinicaltrial.gov NCT07188558) compares the new construct with standard of care (axi-cel or liso-cel) (89). Whether dual-targeting ultimately translates into improved long-term disease control compared with conventional CD19-directed CAR T-cell therapy remains to be established.

### 5.2 CAR products with preserved T-cell stemness

Beyond CAR design, increasing attention has focused on the quality of the infused T-cell product. Accumulating evidence suggests that preserving naïve and stem-cell memory T-cell populations during manufacturing enhances proliferative capacity, persistence, and long-term antitumor activity. Consequently, several next-generation products aim to minimize *ex-vivo* manipulation and enhance *in-vivo* expansion, while enriching for less differentiated T-cell subsets (90). Products such as zamto-cel, ronde-cel, KITE-753, GLPG5101 and rapcabtagene autoleucel (YTB323) incorporate manufacturing strategies designed to preserve stem-like T-cell characteristics (83, 84, 87, 91–93). These approaches include shortened manufacturing times with preservation of early memory T-cell phenotypes (zamto-cel, KITE-753, GLPG5101, YTB323), or specific strategies such as enrichment of CD62L-positive naïve and central memory T cells (ronde-cel) (91). Although based on relatively small cohorts, preliminary studies consistently report favorable expansion kinetics, durable persistence, and manageable toxicity, supporting the hypothesis that optimization of T-cell fitness may be as important as CAR construct design itself.

### 5.3 “Armored” CAR construct and future engineering strategies

A further evolution in CAR T design aims to actively remodel the tumor microenvironment rather than enhancing intrinsic T-cell function. The “armored” CAR T-cells are engineered to constitutively express immunostimulatory molecules capable of overcoming local immune suppression, promoting CAR T expansion, and delaying functional exhaustion (94). HuCART19-IL18 is a fourth-generation anti-CD19 enhanced (“armored”) CAR construct that combines rapid manufacturing (3-day process) with constitutive IL-18 secretion to enhance antitumor immunity and improve CAR T persistence in heavily pretreated patients (94–97). Although clinical experience remains preliminary, these data illustrate the broader concept that future CAR T platforms will increasingly integrate immune modulation with direct cytotoxic activity.

Beyond autologous CAR T-cell products, several alternative cellular platforms are under active investigation, including allogeneic (“off-the-shelf”) CAR T-cells and CAR-natural killer (CAR-NK) cells (98, 99). Allogeneic CAR T-cells seek to improve treatment accessibility by eliminating patient-specific manufacturing, whereas CAR-NK cells may provide effective antitumor activity with a lower risk of severe immune-mediated toxicities. Although several biological and logistical challenges remain, including immune rejection and limited *in vivo* persistence, these next-generation cellular therapies are expected to further expand the therapeutic landscape of LBCL in the coming years (98, 99).

## 6 Discussion

The therapeutic paradigm of R/R LBCL has been profoundly reshaped by the introduction of CD19-directed CAR T-cell therapies. The pivotal phase III ZUMA-7 and TRANSFORM trials established CAR T-cell therapy as the preferred second-line treatment for patients with primary refractory disease or relapse within 12 months after frontline chemoimmunotherapy, fundamentally redefining the role of transplantation. Consequently, auto-HSCT is currently reserved for the favorable subgroup of patients with late relapse (>12 months), in whom durable long-term remissions can still be achieved. In contrast, allo-HSCT has evolved into a highly selected post-CAR salvage strategy for fit patients who achieve disease control after subsequent therapies.

The clinical case presented in this review exemplifies one major unmet need in the CAR T-cell era, such as early relapse with secondary CNS involvement after an initial complete response. Although uncommon, these patients have a particularly poor prognosis and limited therapeutic options. Our patient’s durable remission following salvage allo-HSCT highlights that, despite the expanding availability of novel immunotherapies, allogeneic transplantation continues to represent a potentially curative option in carefully selected individuals.

At the same time, increasing insight into the biological mechanisms underlying CAR T-cell failure, including antigen escape, limited persistence, T-cell exhaustion, and the immunosuppressive TME has driven a new generation of bioengineered cellular therapies. Rather than focusing exclusively on improving response rates, current engineering strategies aim to address these mechanisms directly through dual-target antigen recognition, preservation of T-cell stemness, accelerated manufacturing platforms and modulation of the tumor microenvironment. Looking further ahead, advances in gene editing and *in-vivo* CAR generation may eventually enable universal cellular therapies with simplified manufacturing and broader accessibility, although these approaches remain at an early stage of clinical development.

Future treatment algorithms will probably move beyond the concept of a single “best” CAR construct and instead integrate disease biology, mechanisms of resistance, patient fitness, manufacturing characteristics, and the availability of complementary immunotherapeutic approaches. Ultimately, the future of R/R LBCL management will rely not on replacing “old” cellular therapies with newer platforms, but on integrating these complementary approaches into individualized treatment strategies.
